# EGR1 induces EMT in pancreatic cancer via a P300/SNAI2 pathway

**DOI:** 10.1186/s12967-023-04043-4

**Published:** 2023-03-17

**Authors:** Yuanyang Wang, Cheng Qin, Bangbo Zhao, Zeru Li, Tianyu Li, Xiaoying Yang, Yutong Zhao, Weibin Wang

**Affiliations:** 1 State Key Laboratory of Complex Severe and Rare Diseases, Peking Union Medical College Hospital, Chinese Academy of Medical Science and Peking Union Medical College, Beijing, China; 2 Department of General Surgery, Peking Union Medical College Hospital, Chinese Academy of Medical Science and Peking Union Medical College, Beijing, China

**Keywords:** EGR1, Pancreatic cancer, EMT, SNAI2, Gene transcription, p300/CBP, Cancer metastasis

## Abstract

**Background:**

The prognosis of pancreatic cancer patients remains relatively poor. Although some patients would receive surgical resection, distant metastasis frequently occurs within one year. Epithelial-mesenchymal transition (EMT), as a pathological mechanism in cancer progression, contributed to the local and distant metastasis of pancreatic cancer.

**Methods:**

Tissue microarray analysis and immunohistochemistry assays were used to compare the expression of EGR1 in pancreatic cancer and normal pancreatic tissues. Transwell chambers were used to evaluated the migration and invasion ability of cancer cells. Immunofluorescence was utilized to assess the expression of E-cadherin. ChIP-qPCR assay was applied to verify the combination of EGR1 and SNAI2 promoter sequences. Dual-luciferase reporter assay was used to detect the gene promoter activation. Co-IP assay was conducted to verify the interaction of EGR1 and p300/CBP.

**Results:**

EGR1 was highly expressed in pancreatic cancer rather than normal pancreatic tissues and correlated with poor prognosis and cancer metastasis. EGR1 was proved to enhance the migration and invasion ability of pancreatic cells. Besides, EGR1 was positively correlated with EMT process in pancreatic cancer, via a SNAI2-dependent pathway. P300/CBP was found to play an auxiliary role in the transcriptional activation of the SNAI2 gene by EGR1. Finally, in vivo experiments also proved that EGR1 promoted liver metastasis of pancreatic cancer.

**Conclusion:**

Our findings implied the EMT-promoting effect of EGR1 in pancreatic cancer and revealed the intrinsic mechanism. Blocking the expression of EGR1 may be a new anticancer strategy for pancreatic cancer.

**Graphical Abstract:**

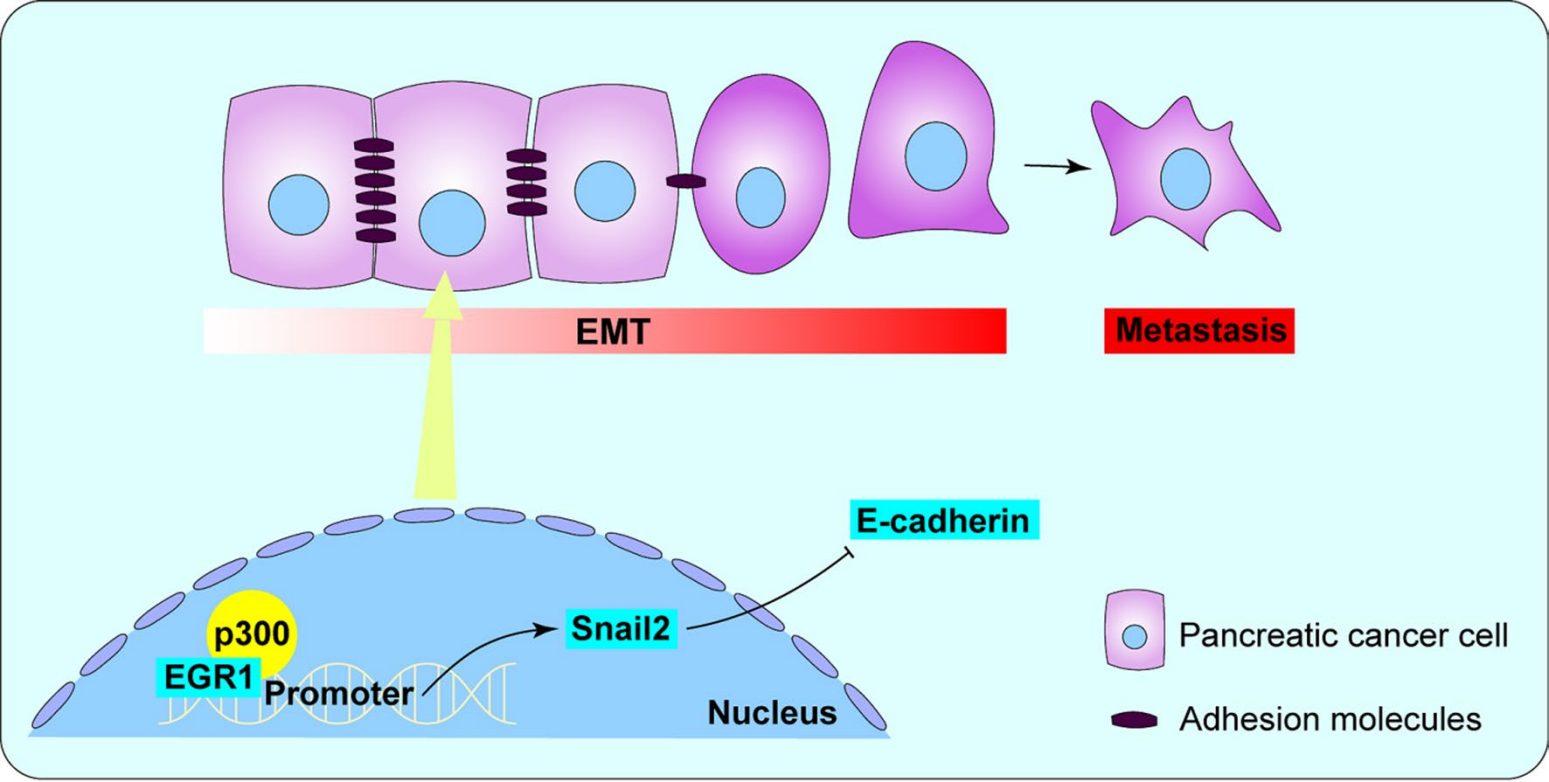

**Supplementary Information:**

The online version contains supplementary material available at 10.1186/s12967-023-04043-4.

## Introduction

Pancreatic cancer is one of the deadliest malignant diseases, with a 5-year survival rate of 11% in all stages [1]. Surgical operation is the only way to cure the disease, however, less than 20% of the patients are advisable for surgery and most of the patients are diagnosed at a late metastatic stage [2]. However, more than 60% of the patients with pancreatic cancer who underwent surgery developed distant metastasis within 24 months after surgery [3]. At present, the treatment options for pancreatic cancer are very limited, so more constructive and effective interventions against tumor metastasis are urgently needed.

Epithelial-mesenchymal transition (EMT) refers to a physiological mechanism for the development and remodeling of cells and tissue, as well as a pathological mechanism in cancer progression, during which tumor cells lose their characteristics of polarization and obtain mesenchymal features [4]. The initiation of tumor metastasis requires cell migration and invasion, which is realized by EMT [5]. A typical feature of the EMT process is the loss of expression of intercellular adhesion molecules, such as E-cadherin and the gain of mesenchymal markers, such as N-cadherin, Vimentin, etc. [6, 7]. Previous studies have proved that the EMT process contributed to the high malignancy of pancreatic cancer [8].

Early growth response 1 (EGR1) serves as a member of the EGR family, which could be stimulated by serum, growth factors, cytokines, or other stress stimulation [9, 10]. As a transcription factor, EGR1 takes effects through promotion or inhibition the transcription of its downstream genes. The role of EGR1 in cancers is diverse. In non-small cell lung cancer, EGR1 promoted ionizing radiation-induced EMT via the Egr-1/cathepsin L pathway [11]. In prostate cancer, EGR1 also accelerated cancer metastasis PI3K/PTEN/Akt axis [12]. However, in some types of cancers, such as human hepatocarcinoma, leukemia, human fibrosarcoma, etc. EGR1 suppressed tumor proliferation and invasion ability [13–15].

In pancreatic cancer, whether EGR1 acts as a tumor promoter or tumor suppressor has not been determined. In our current study, we firstly reported the evidence that EGR1 promoted pancreatic cancer migration and invasion. Mechanically, EGR1 interacted with p300/CBP and combined to the promoter region of SNAI2, thus restrained E-cadherin expression and promoted cancer metastasis.

## Material and methods

### Cell culture and reagents

Human pancreatic cancer cell lines (PANC1 and BxPC-3) were bought from the American Type Culture Collection and cultured in Dulbecco’s Modified Eagle Medium (DMEM, Gibco^™^, ThermoFisher Scientific Inc., Waltham, Massachusetts, USA) or Roswell Park Memorial Institute Medium (RPMI, Corning, NY, USA) respectively with 10% fetal bovine serum at 37 ℃ under 5% CO_2_. Penicillin (100 U/ml) and streptomycin (100 µg/ml) (Biological Industries, Israel) were added to the medium. Selective CBP/EP300 inhibitor GNE-272 was bought from MedChemExpress (MCE, New Jersey, USA). Sulforhodamine B (SRB) was bought from Sigma-Aldrich (Merck KGaA, Darmstadt, Germany).

### RNA extraction and quantitative real-time PCR (qRT-PCR)

Total RNA from cells was isolated by Trizol reagent (Thermo Scientific™, Waltham, Massachusetts, USA) and reverse transcription of RNA was conducted with RevertAid First Strand cDNA Synthesis Kit (Thermo Scientific™, Waltham, Massachusetts, USA) following the instructions. The PowerUp™ SYBR™ Green Master Mix (Applied Biosystems™, ThermoFisher Scientific Inc., Waltham, Massachusetts, USA) was used to perform Quantitative Real-Time PCR. β-actin was selected as an internal reference gene and fold enrichment was confirmed by calculating the ΔΔCt value. The primers used in this research were listed in Additional file 1: Table S1.

### Cell transfection and lentiviral infection

3 × 10^5^ cells were placed in each well of a 6-well plate. On the next day, transfection of siRNA or plasmids was conducted with Lipofectamine™ 3000 (Thermo Scientific™, Waltham, Massachusetts, USA) following the protocols. Lentiviral transductions were performed to construct stable EGR1-overexpression or EGR1-knockdown cell lines. 2 × 10^5^ cells were prepared for transduction and infected by EGR1-overexpression or EGR1-knockdown lentiviruses (Shanghai Genechem) at a multiplicity of infection (MOI) of 10. After 72 h of infection, the cells were cultured in a medium containing 2 mg/L puromycin for 2 weeks to remove non-infected cells.

### Cell migration and invasion assay

Transwell BD Chamber (8 μm, Corning, NY, USA) was used to perform migration and invasion assay. In brief, 4 × 10^4^ cells in 100 μl serum-free medium were seeded into the upper chamber and 600 μl medium containing 10% FBS was put in the lower chamber in each well of a 6-well plate. After incubation for 24 h at 37 ℃ under 5% CO_2_, the cells were fixed with methanol and dyed with hematoxylin and eosin. Three random fields were photographed under a microscope (objective lens: tenfold; eyepiece: tenfold). For the invasion experiment only, 40 μl BD Matrigel (356234, Corning, NY, USA) was used to cover the bottom of the upper chamber.

### Wound healing assays

Wound healing assays were performed to test the migration ability in vitro. Briefly, 6 × 10^5^ cells within 70 μl medium containing 10% FBS were planted in the each well of the ibidi Culture-Insert (ibidi, Germany) in a well of the 24-well plate. After cell adhesion, the insert was removed and the medium was changed to serum-free medium. The cells were washed and photographed at each time point by a microscope.

### Western blotting assay and antibodies

RIPA lysis buffer (P0013B, Beyotime, China) was used to extract proteins from cells. BCA Protein Assay Kit (P0012, Beyotime, China) was used to measure the concentration of proteins. Each cell protein lysates were mixed with 5 × SDS buffer and boiled at 100 ℃ for 10 min before western blotting. The proteins were separated in sodium dodecyl sulfate–polyacrylamide gel electrophoresis and transferred to the polyvinylidene difluoride (PVDF) membrane. Then, the PVDF membrane was blocked in 5% milk in tris buffered saline with 0.05% Tween 20 (TBST) for 90 min. The membranes were then incubated with primary antibodies at 4 ℃ overnight. The membranes were washed by TBST and incubated with second antibodies for 60 min and washed by TBST again. The proteins on the PVDF membrane were then visualized by an enhanced chemiluminescence detection kit (Beyotime, China) and an automatic chemiluminescence image analysis system (Tanon, China). The protein bands were further quantified by Image J software. The primary antibodies used in the study were: anti-E-cadherin (20874-1-AP, Proteintech, China), anti-N-cadherin (ab18203, Abcam, USA), anti-vimentin (VIM) (10366-1-AP, Proteintech, China), anti-SNAI2 (12129-1-AP, Proteintech, China), anti-β-actin (A1978, MilliporeSigma, USA), anti-EGR1 (ab194357, Abcam, USA), anti-KAT3B/p300 (ab275378, Abcam, USA), anti-CREBBP (CBP) (ab253202, Abcam, USA).

### Tissue microarray analysis and immunohistochemistry (IHC) assay

Tissue microarray of pancreatic cancer tissues and adjacent normal pancreatic tissues was bought from SUPERBIOTEK (Shanghai, China). Immunohistochemistry (IHC) assay was performed to explore the expression of EGR1 in this tissue microarray and anti-EGR1 (ab194357, Abcam, USA) was used as the primary antibody. The scores of the stained microarray were independently evaluated by two pathologists. The areas with almost no stained cells were considered negative (proportion score 0). The areas with less than 25% stained cells were considered weakly positive (proportion score 1). The areas with less than 50% but more than 25% stained cells were considered weakly positive (proportion score 2). The areas with less than 75% but more than 50% stained cells were considered positive (proportion score 3). The areas with more than 75% stained cells were considered strongly positive (proportion score 4). Primary non-stained particles were considered negative (staining intensity 0). Lightly yellow particles were considered low intensity (staining intensity 1). Brownish-yellow particles were considered moderate intensity (staining intensity 2). Brown particles were considered high intensity (staining intensity 3). The staining fraction is equal to the proportion of tumor cells multiplied by the staining intensity. The staining scores of 0, 1, 2, 3, 4 were defined as the low EGR1 expression group. The staining scores between 6, 8, 9, 12 were defined as the high EGR1 expression group.

### Immunofluorescence (IF) assay

Immunofluorescence (IF) assay was performed to visualize the expression of proteins. Anti-E-cadherin (20874-1-AP, Proteintech, China) was used as the first antibody. Alexa Fluor 488 (A0428, Beyotime, China) was used as the secondary antibody. In brief, 2 × 10^4^ transfected cells were seeded in a chamber of the 8 Chambered cover glass (C8-1-N, Cellvis, USA). After the cell attachment, the cells were fixed with 4% paraformaldehyde for 15 min. 3% BSA solution was used to seal off for 30 min at the temperature of 37 ℃. Then the cells were incubating with primary antibody overnight at the temperature of 4 ℃ and with secondary antibody for 45 min at the temperature of 37 ℃. At last, DAPI (C1002, Beyotime, China) was used to stain the nucleus. The immunofluorescence was detected and photographed by a laser confocal microscope (AX, Nikon, Japan).

### Coimmunoprecipitation (Co-IP) assay

Pierce^™^ Crosslink Magnetic IP/Co-IP Kit (88805, Thermo Scientific^™^, Waltham, Massachusetts, USA) was used in this experiment. In brief, protein A/G magnetic beads were bound and crosslinked by DSS to the first antibody. Then, the cell lysates were incubated with the beads overnight. Finally, the bound antigens were eluted and subsequently analyzed by Western blotting. Anti-EGR1 (ab194357, Abcam, USA) and anti-KAT3B/p300 (ab275378, Abcam, USA) were used as the first antibody.

### Chromatin-immunoprecipitation (ChIP) assay

Pierce™ Magnetic ChIP Kit (26157, Thermo Scientific™, Waltham, Massachusetts, USA) was used in this experiment. The cells were cultivated in 10 cm dishes and collected after being fixed with formaldehyde. Then the cell membrane and cytosol were lysed and the nucleic acids were digested by MNase. The chromatins with proteins were further obtained by sonication. Anti-IgG and anti-EGR1 (ab194357, Abcam, USA) were used as the antibody to incubate with chromatins. Protein A/G magnetic beads were used to purify the DNA. The results were further analyzed by qRT-PCR.

### Dual-luciferase reporter assay

Dual-luciferase reporter assay was performed to detect the gene promoter activation. The control lentivirus-CON254 and lentivirus-EGR1 cell lines were constructed to perform dual-luciferase reporter assay. The cells were seeded in 6-well plates and then transfected with wild-type or mutant human EGR1-promoter-luciferase reporter. After 48 h transfection, the cells were lysed and performed a dual-luciferase reporter assay following the instruction of the Dual Luciferase Reporter Gene Assay Kit (Yeasen Biotechnology, Shanghai). The bioluminescence was detected by a multifunctional microplate reader.

### In vivo* experiment*

Normal control and EGR1-overexpressed PANC1 cells were constructed via stable transfection of the aforementioned lentivirus. The model of liver metastasis of pancreatic cancer was established by injecting cancer cells (1 × 10^6^ cells/50 μL) to the spleen of BALB/c-nude mice (6 weeks, male). Each group contained twelve mice and the mice were sacrificed after four weeks. The livers were collected for visual observation and hematoxylin–eosin (HE) staining. The metastatic colonies were then identified and counted under a light microscope. Our experiments were performed under the guidelines of Peking Union Medical College Ethical Committee (Beijing, China).

### Statistical analysis

The data were presented as means with standard error. Student t-tests were used to compare the statistical difference. P < 0.05 was considered statistical significance. All experiments were performed at least 3 times. The charts and images were drawn by the Prism 8 software.

## Results

### The expression and clinicopathological features of EGR1 in pancreatic cancer tissues

**Fig. 1:**
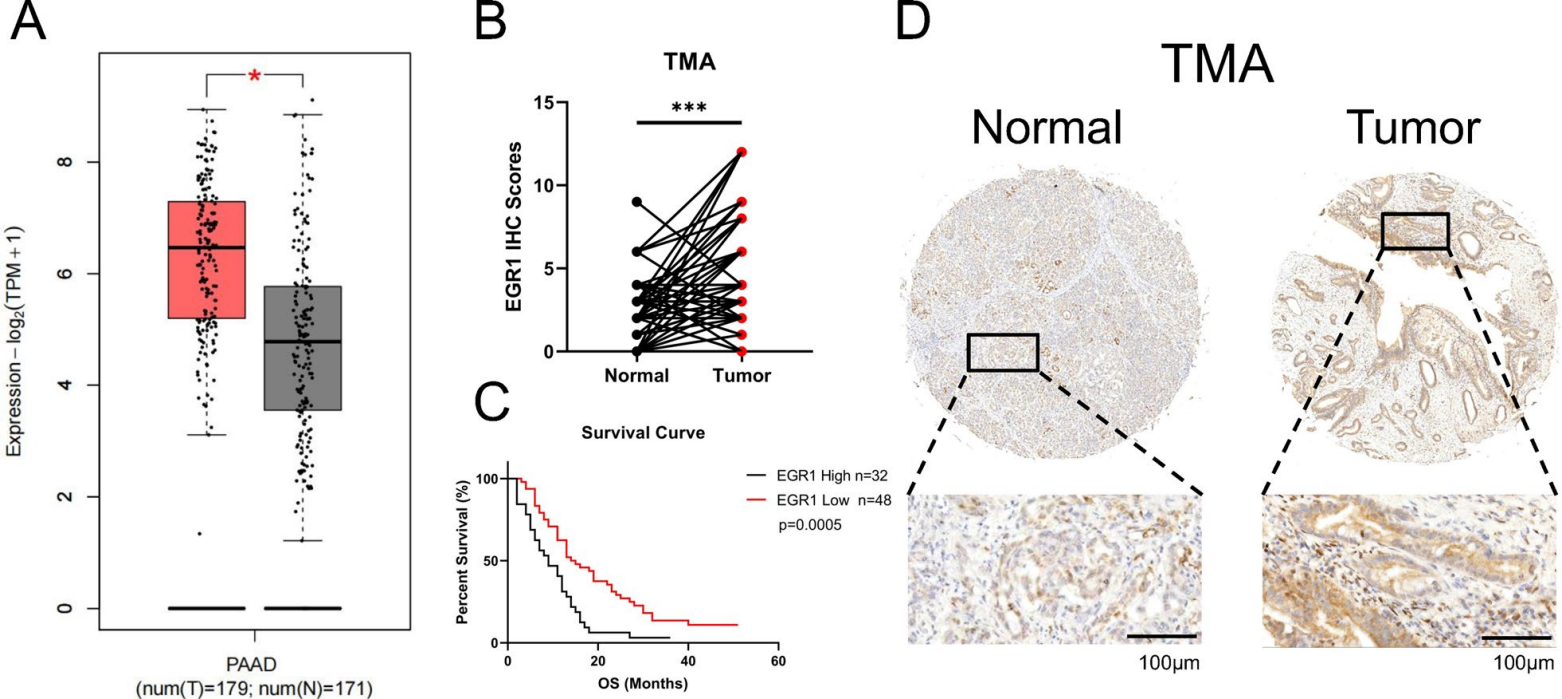
The expression and clinicopathological features of EGR1 in pancreatic cancer tissues. **A** The expression of EGR1 in TCGA and GTEx databases. **B** The protein expression of EGR1 in pancreatic cancer tissues and paired adjacent normal pancreatic tissues displayed by IHC scores. **C** The survival curve of EGR1 in TMA. **D** Representative IHC images of EGR1 in pancreatic cancer tissues and normal pancreatic tissues. Scale bars: 100 μm. ***: p < 0.001

We firstly analyzed the expression of EGR1 in TCGA and GTEx databases. The results showed that EGR1 was highly expressed in pancreatic cancer compared with normal pancreatic tissue (Fig. 1A). Further, a Tissue Microarray (TMA) was performed to investigate the relationship between EGR1 protein expression and the clinicopathological characteristics of patients with pancreatic cancer. The IHC staining indicated excessive EGR1 expression in pancreatic cancer tissues compared with paired adjacent normal pancreatic tissues (Fig. 1B). The Kaplan–Meier survival curve was drawn, showing that patients with high expression of EGR1 (score 0, 1, 2, 3, 4) had poor overall survival (OS) compared with the patients with low EGR1 expression (score 6, 8, 9, 12) in the TMA (p = 0.0005) (Fig. 1C). The EGR1 expression in normal pancreatic ducts was significantly lower than in pancreatic ductal adenocarcinoma (Fig. 1D). Besides, we found patients with higher expression of EGR1 were correlated with higher tumor N stage (p < 0.05) and TNM stage (p < 0.05) (Table 1), indicating the tumor-promoting role of EGR1. By univariate COX regression analysis, we found Differential degree, T stage, N stage, TNM stage and EGR1 expression were the significant risk factors affecting the survival rate of patients (p < 0.05) (Table 2). In the multivariate Cox analysis, Differential degree, TNM stage and EGR1 expression were confirmed associated with the survival of patients (p < 0.05) (Table 2). These results indicated that EGR1 that highly expressed in pancreatic cancer was correlated with higher tumor stage and poor prognosis.

**Table 1:** Correlations of EGR1 expression levels with clinical and pathologic parameters

Variables	n	EGR1 expression		P value
		Low group n = 48	High group n = 32	
Gender				0.405
Male	41	24	17	
Female	39	24	15	
Age				0.785
< 60	32	21	11	
≥ 60	48	27	21	
Location				0.195
Head	48	26	22	
Body/tail	32	22	10	
Differential degree				0.413
Low	43	24	19	
High/moderate	37	24	13	
T stage				0.142
T1/T2	55	36	19	
T3/T4	25	12	13	
N stage				0.001
N0	50	37	13	
N1/N2	30	11	19

**Table 2:** Univariate and multivariate Cox analysis of possible risk prognostic factors

Variables	n	Univariate		Multivariate	
		HR (95% CI)	P value	HR (95% CI)	P value
Gender			0.177		
Male	41	1			
Female	39	0.726(0.456–1.156)			
Age			0.871		
< 60	32	1			
≥ 60	48	0.962(0.602–1.536)			
Location			0.450		
Head	48	1			
Body/tail	32	0.834(0.529–1.339)			
Differential degree			0.000		0.000
Low	43	1		1	
High/moderate	37	2.657(1.650–4.276)		2.871(1.733–4.758)	
T stage			0.046		0.307
T1/T2	55	1		1	
T3/T4	25	1.695(1.025–2.803)		1.367(0.751–2.490)	
N stage			0.001		0.543
N0	50	1		1	
N1/N2	30	2.384(1.439–3.952)		0.779(0.348–1.744)	
TNM stage			0.000		0.037
I/IIA	41	1		1	
IIB/III/IV	39	3.108(1.834–5.265)		2.631(1.059–6.532)	
EGR1 expression			0.001		0.037
Low	48	1		1	
High	32	2.246(1.382–3.648)		1.79(1.036–3.092)	

### EGR1 enhanced pancreatic cancer migration and invasion ability

**Fig. 2:**
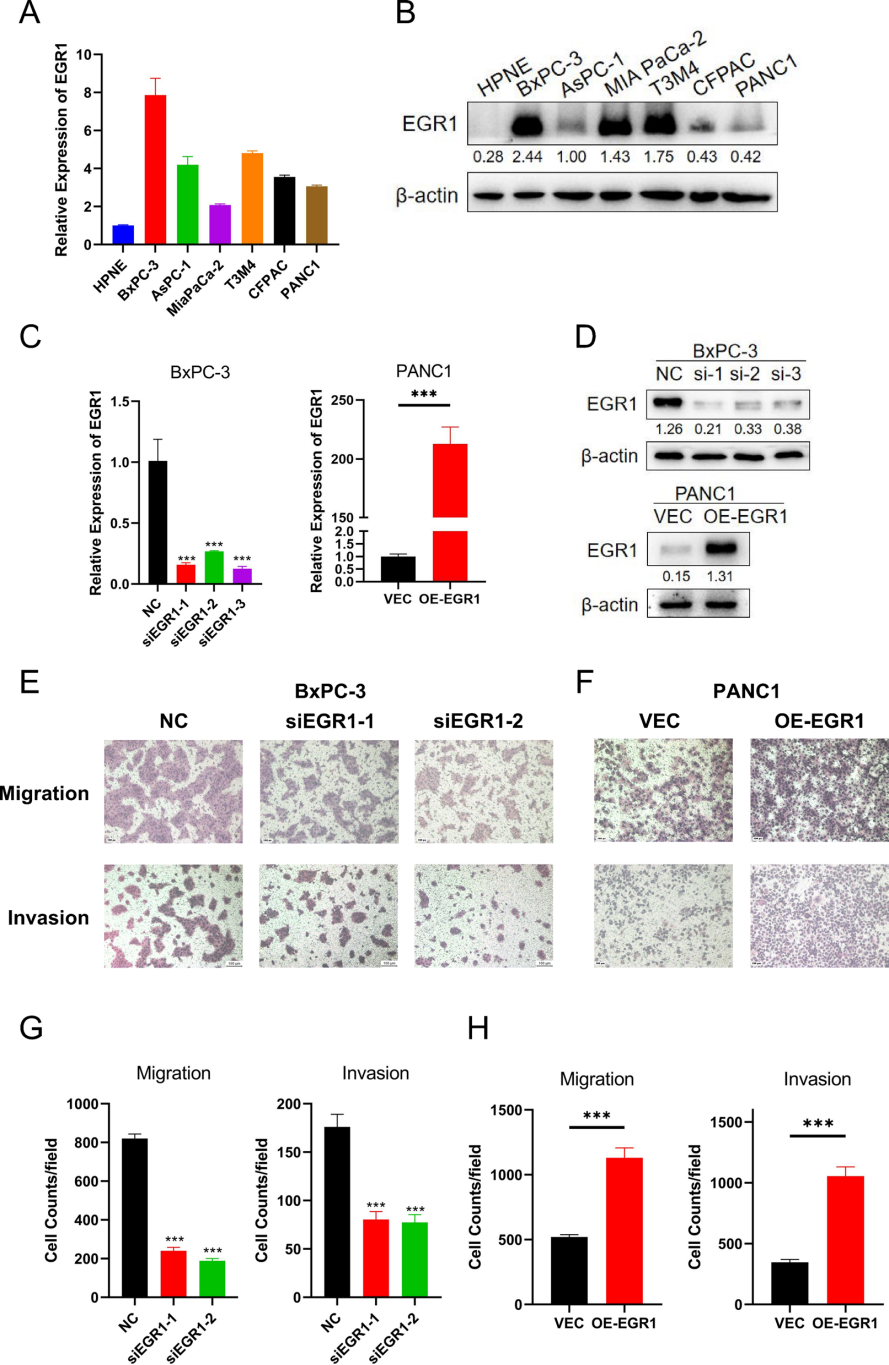
EGR1 enhanced pancreatic cancer migration and invasion ability. **A** The mRNA expression of EGR1 in different pancreatic cancer cell lines were shown. **B** The protein expression of EGR1 in different pancreatic cancer cell lines were shown. **C** The effect of EGR1 knockdown and EGR1 overexpression was verified by qPCR method. **D** The effect of EGR1 knockdown and EGR1 overexpression verified by western blot assay. **E** and **G** The cell migration and invasion ability were downregulated after knockdown of EGR1 in BxPC-3. **F** and **H** The cell migration and invasion ability were enhanced after overexpression of EGR1 in PANC1. ***: p < 0.001

To investigate the function of EGR1 in vitro, we firstly detected the expression level of EGR1 in different cell lines. The results of qRT-PCR and western blot showed that low expression of EGR1 in normal pancreatic cell HPNE (1 $\pm$ 0.036) and excessive expression of EGR1 (4.261 $\pm$ 2.001) in cancer cells (Fig. 2A, B). Statistically significant differences of EGR1 expression were found between each two cell lines in both experiments. Considering the results of western blot, BxPC-3 cell line with high EGR1 expression and PANC1 cell line with low EGR1 expression were selected to perform temporarily knockdown or overexpression of EGR1. The efficiencies were detected by qPCR and western blot and all three siRNA and overexpression plasmids showed good effects (Fig. 2C, D). Further, the migration and invasion ability of pancreatic cancer cells were detected after knockdown or overexpression of EGR1. The migrated or invaded cells were significantly decreased after knockdown of EGR1 (820.000 $\pm$ 24.000 vs. 240.000 $\pm$ 18.330; p < 0.05 or 820.000 $\pm$ 24.000 vs. 188.333 $\pm$ 11.676; p < 0.05) (176.000 $\pm$ 13.115 vs. 80.333 $\pm$ 8.327; p < 0.05 or 176.000 $\pm$ 13.115 vs. 77.333 $\pm$ 8.021; p < 0.05) (Fig. 2E, G). Similarly, the migrated or invaded cells were significantly increased after overexpression of EGR1 (520.333 $\pm$ 17.559 vs. 1130.000 $\pm$ 77.175; p < 0.05) (347.333 $\pm$ 22.121 vs. 1054.667 $\pm$ 76.559; p < 0.05) (Fig. 2F, H). Wound healing assay was also performed to verify the migration ability of cancer cells after knockdown or overexpression of EGR1 (Additional file 2: Fig. S1A, B). These results uncovered that EGR1 promoted pancreatic cancer migration and invasion ability.

### EGR1 promoted EMT process in pancreatic cancer cell

**Fig. 3:**
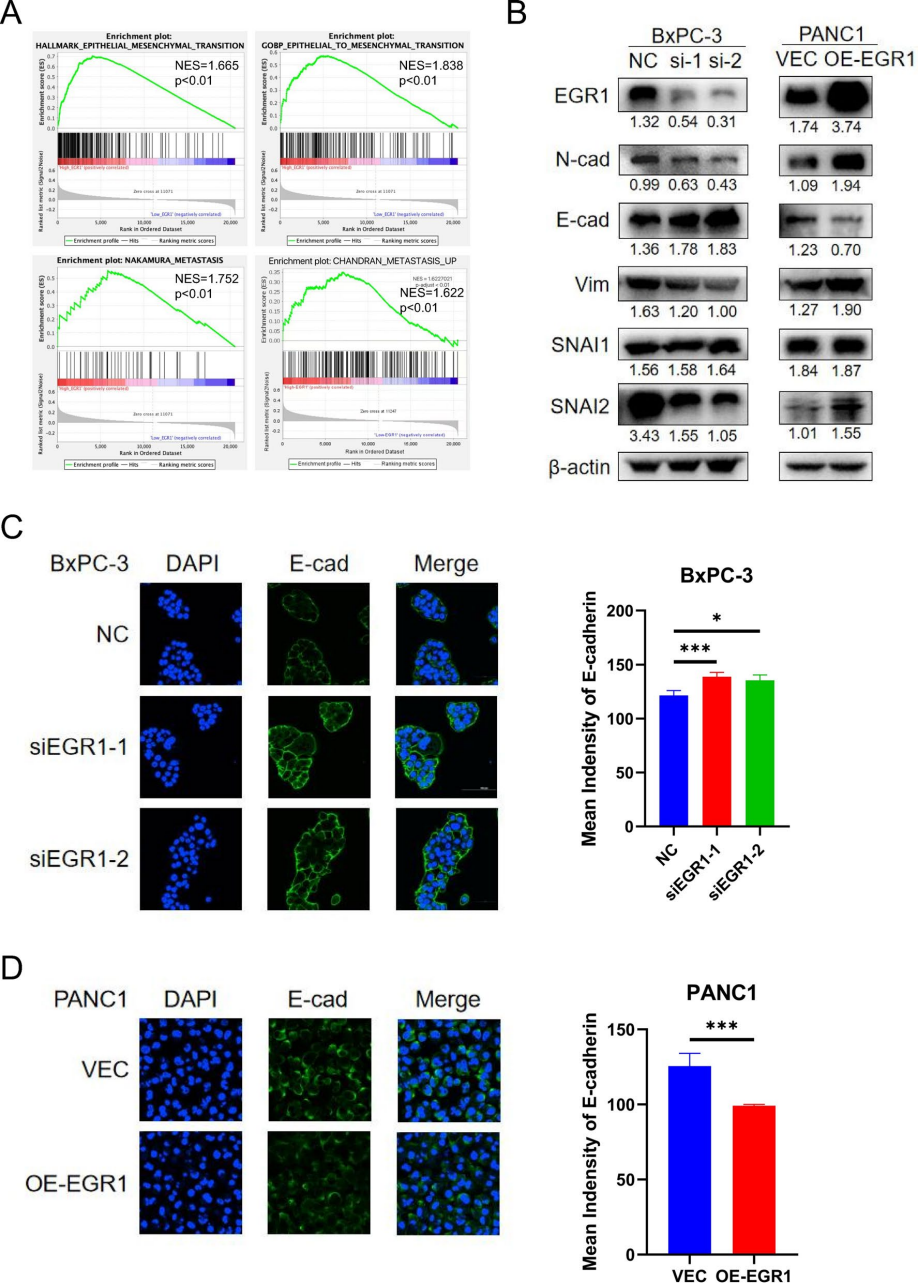
EGR1 promoted EMT process in pancreatic cancer cell. **A** The GSEA analysis showed that EGR1 was correlated with metastasis and EMT pathway in pancreatic cancer. **B** The expression of EMT-related protein after knockdown or overexpression of EGR1. **C** Representative images of immunofluorescence staining showed the fluorescence of E-cadherin and nucleus after knockdown or overexpression of EGR1. **D** The statistical column chart of the fluorescence intensity of E-cadherin. ***: p < 0.001

To further investigate the reason why EGR1 affected the migration and invasion ability of pancreatic cancer cell, we obtained the RNA expression profiles of pancreatic cancer tissues from TCGA database (https://portal.gdc.cancer.gov/) and performed Gene Set Enrichment Analysis (GSEA) analysis based on the expression of EGR1. We found that high EGR1 expression was correlated with HALLMARK_EPITHELIAL_MESENCHYMAL_TRANSITION (NES = 1.665, p < 0.01), GOBP_EPITHELIAL_MESENCHYMAL_TRANSITION (NES = 1.838, p < 0.01), NAKAMURA_METASTASIS (NES = 1.752, p < 0.01) and CHANDRAN_METASTASIS_UP (NES = 1.622, p < 0.01) pathway (Fig. 3A). Thus, we wondered whether EGR1 was involved in the EMT process in pancreatic cancer. By immunoblotting analysis, we found that the expression of N-cadherin, Vimentin and SNAI2 were significantly downregulated and the expression of E-cadherin was significantly upregulated after knockdown of EGR1 while the expression of SNAI1 had no difference in BxPC-3 (Fig. 3B; Additional file 2: Fig. S1C). The expression of N-cadherin, Vimentin and SNAI2 were significantly upregulated and the expression of E-cadherin was significantly downregulated after overexpression of EGR1 while the expression of SNAI1 had no difference in PANC1 (Fig. 3B; Additional file 2: Fig. S1C). Besides, IF assay was performed to visualize the expression of E-cadherin and we found the fluorescence intensity of E-cadherin was upregulated after knockdown of EGR1 in BxPC-3 and downregulated after overexpression of EGR1 in PANC1 (Fig. 3C, D).

### SNAI2 was a direct target of EGR1 in pancreatic cancer

**Fig. 4:**
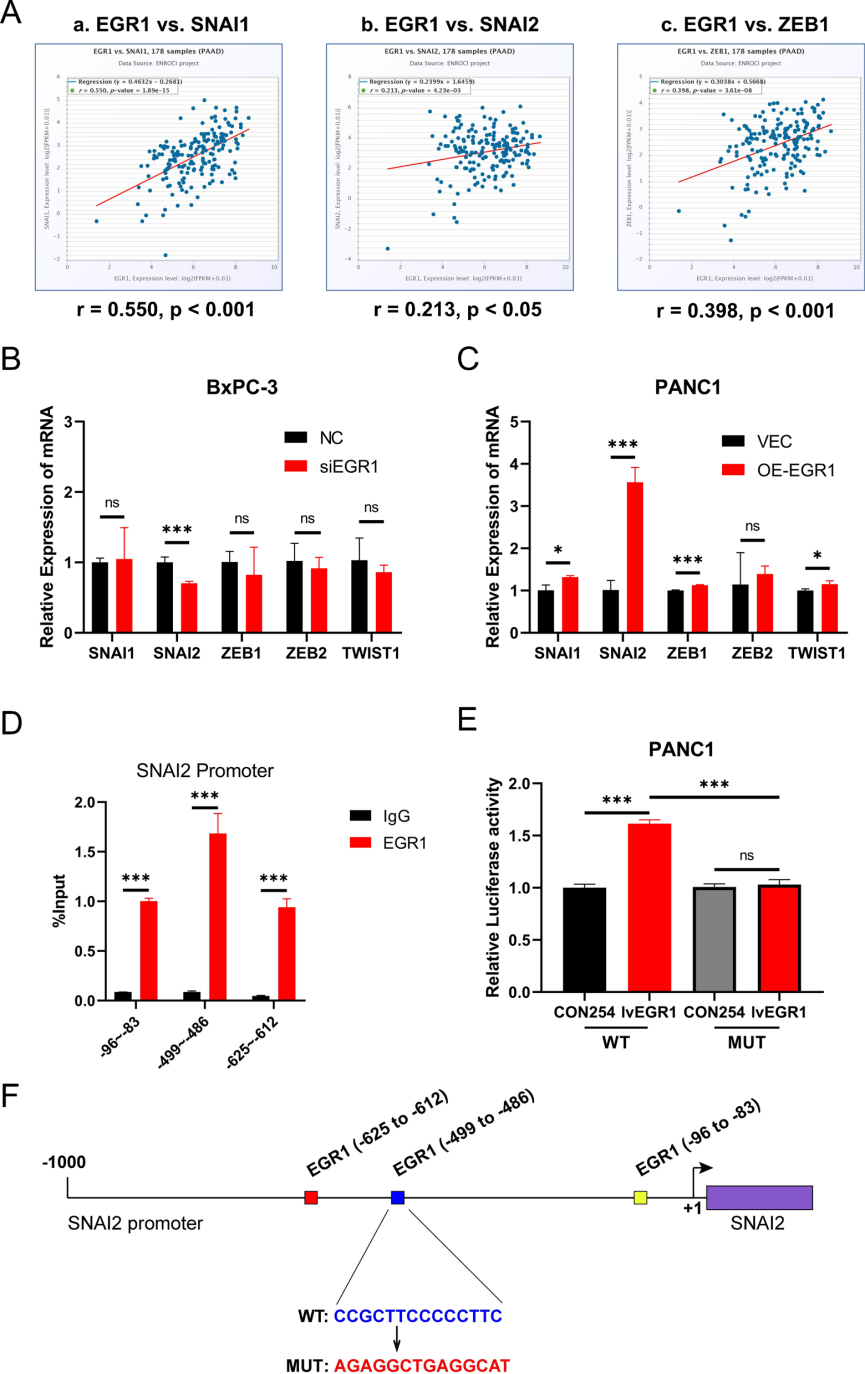
SNAI2 was a direct target of EGR1 in pancreatic cancer. **A** The predicted RNA correlation of EGR1 and SNAI1, SNAI2, ZEB1 in pancreatic cancer. **B** and **C** QPCR analysis showed the relative expression of the above genes after knockdown and overexpression of EGR1. **D** ChIP-qPCR assay showed the binding capability of EGR1 with different predicted binding sites. **E** Dual-luciferase reporter assay indicated the promoter activity of SNAI2 encoding wild-type (WT) or mutant EGR1-binding site (MUT). **F** A schematic depicting EGR1 bound with SNAI2 promoter to activate SNAI2 transcription. ***: p < 0.001

To further clarify the mechanism by which EGR1 regulated the EMT pathway, we wondered whether the EMT-related transcription factors were regulated by EGR1. Gene correlation analysis were performed based on the RNA data from the TCGA database via GEPIA2 website (GEPIA 2 (cancer-pku.cn)). The RNA expression of EGR1 was positively related with the RNA expression of SNAI1 (a), SNAI2 (b), ZEB1 (c), ZEB2 (d) and TWIST1 (e) (Fig. 4A; Additional file 2: Fig. S1D). However, by qRT-PCR analysis, only the expression of SNAI2 was downregulated after knockdown of EGR1 in BxPC-3 and significantly upregulated after overexpression of EGR1 in PANC1 (Fig. 4B, C). The expression of SNAI1 was not changed in BxPC-3 but slightly upregulated in PANC1. Thus, we designed primers according to the predicted combining site of EGR1 on the SNAI2 promoter by JASPAR database (JASPAR—A database of transcription factor binding profiles (genereg.net)) (showed in Additional file 1: Table S1) and performed ChIP-qPCR analysis. The results showed that EGR1 had stronger binding ability to the DNA sequences from the -499 to -486 positions of SNAI2 than other two binding sites (− 96 to − 83 or − 625 to − 612) (Fig. 4D). To elucidate the role of SNAI1, ChIP-qPCR analysis on SNAI1 promoter was also performed and there was no difference between IgG group and EGR1 group (Additional file 2: Fig. S1E). Further, dual-luciferase reporter gene was constructed to detect the SNAI2 promoter activity. The results showed that in the WT-promoter group, the relative luciferase activity was upregulated after overexpression of EGR1 but unchanged in the MUT-promoter group (Fig. 4E). These results indicated that EGR1 was directly bound with the SNAI2 promoter at the specific DNA sequences to activate the SNAI2 transcription (Fig. 4F).

### EGR1 promoted pancreatic cancer migration and invasion via SNAI2-dependent EMT pathway

**Fig. 5:**
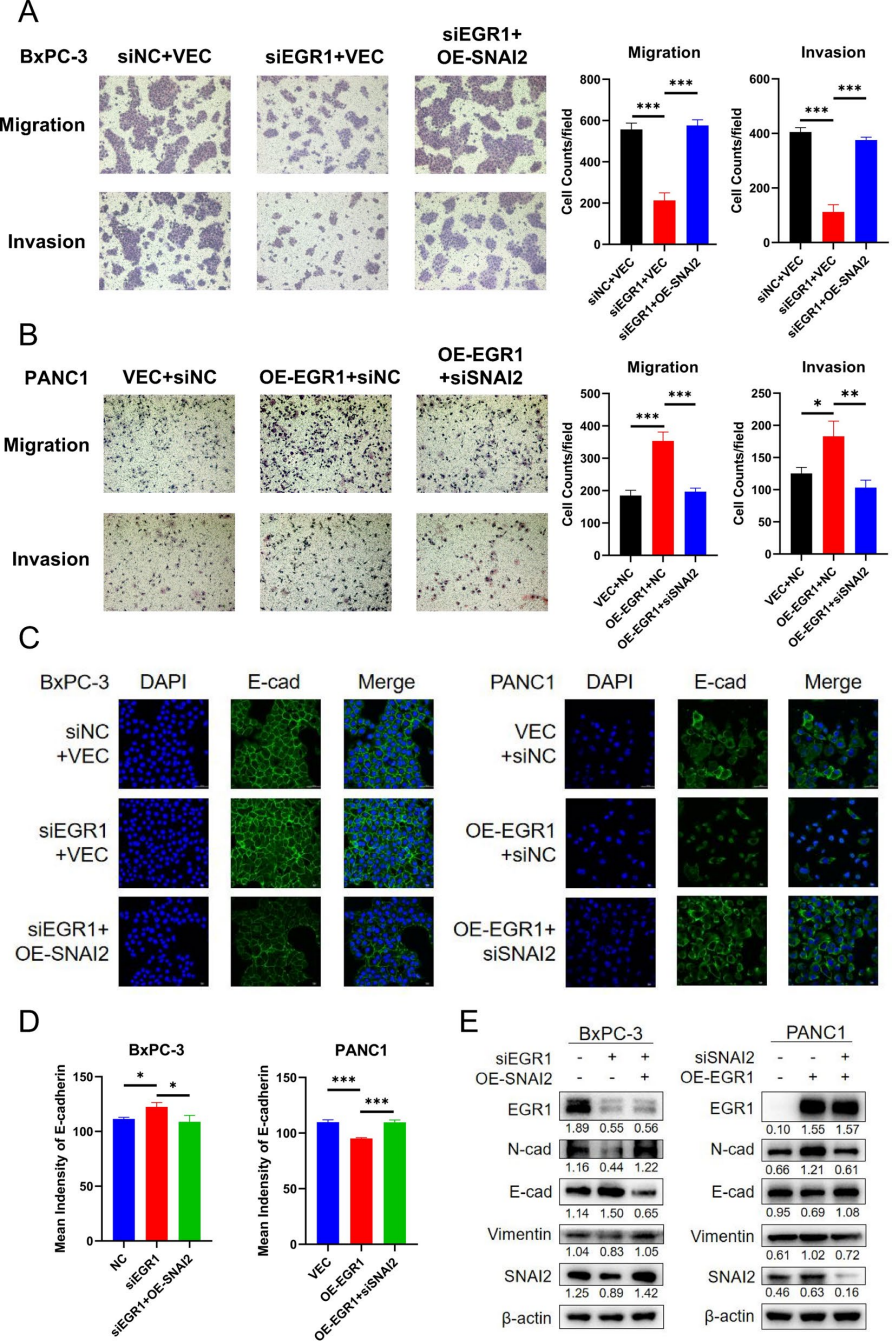
EGR1 promoted migration and invasion ability via SNAI2-dependent EMT pathway in pancreatic cancer cells. **A** Overexpression of SNAI2 offset the effect of EGR1 knockdown on cell migration and invasion ability. **B** Knockdown of SNAI2 offset the effect of EGR1 overexpression on cell migration and invasion ability. **C** Representative images of immunofluorescence staining showed the fluorescence of E-cadherin and nucleus after corresponding processing. **D** Column chart of the fluorescence intensity of E-cadherin corresponding to figure **C**. **E** The expression of EMT-related proteins by western blot after EGR1 knockdown and SNAI2 overexpression in BxPC-3 or EGR1 overexpression and SNAI2 knockdown in PANC1. *: p < 0.05; ***: p < 0.001

SNAI2 (Snail2/Slug) is an EMT-related transcription factor and functions as a E-cadherin transcription repressor [16]. We thus wondered whether the effect of EGR1 on EMT-pathway was dependent on SNAI2. SNAI2 overexpression and knockdown were performed independently in BxPC-3 and PANC1 and the efficiency were shown in the Additional file 2: Fig. S1F. The effect of SNAI2 was then tested and verified in pancreatic cancer. The cell migration and invasion ability were downregulated after knockdown of SNAI2 in BxPC-3 and upregulated after overexpression of SNAI2 in PANC1 (Additional file 3: Fig. S2A, B). Further, the mean immunofluorescence intensity of E-cadherin was upregulated after knockdown of SNAI2 in BxPC-3 and downregulated after overexpression of SNAI2 in PANC1 (Additional file 3: Fig. S2C). These results indicated SNAI2 promoted metastasis and repressed the expression of E-cadherin. Then the rescue experiments were performed. In BxPC-3, after knockdown of EGR1, the cell migration and invasion ability were downregulated and after further overexpression of SNAI2, the cell migration and invasion ability were recovered (Fig. 5A). In PANC1, after overexpression of EGR1, the cell migration and invasion ability were upregulated and after further knockdown of SNAI2, the cell migration and invasion ability were returned to the baseline (Fig. 5B). Further, IF was performed to visualize the expression of E-cadherin. In BxPC-3, the E-cadherin expression was upregulated after knockdown of EGR1 and downregulated after further overexpression of SNAI2 (Fig. 5 C). In PANC1, the E-cadherin expression was downregulated after overexpression of EGR1 and recovered after further knockdown of SNAI2 (Fig. 5C). The relative immunofluorescence intensity of E-cadherin was calculated (Fig. 5D). Western blot images showed in BxPC-3 the expression of N-cadherin, Vimentin and SNAI2 were downregulated after EGR1 knockdown and upregulated after SNAI2 overexpression while E-cadherin changed in the opposite direction (Fig. 5E). And in PANC1 the expression of N-cadherin, Vimentin and SNAI2 were upregulated after EGR1 overexpression and downregulated after SNAI2 knockdown while E-cadherin changed in the opposite direction (Fig. 5E). These results indicated that EGR1 promoted pancreatic cancer migration and invasion and regulated EMT pathway by controlling SNAI2 expression.

### P300/CBP functioned as a transcriptional coactivator in the EGR1-SNAI2 regulation

**Fig. 6:**
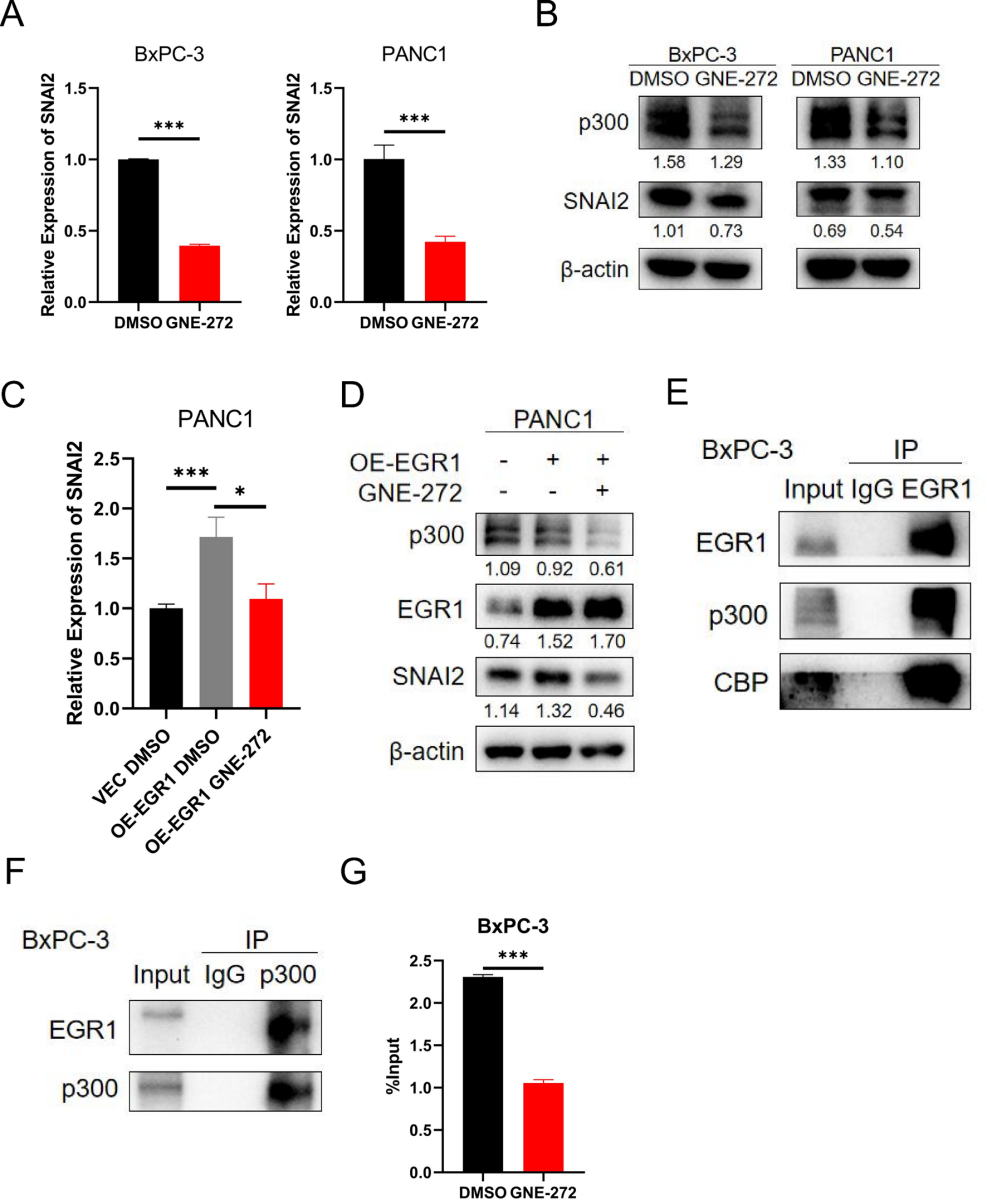
EGR1 interacted with p300/CBP to activate SNAI2 transcription. **A** The relative mRNA expression of SNAI2 was downregulated after treatment with GNE-272 in BxPC-3 and PANC1. **B** The expression of p300 and SNAI2 protein were downregulated after treatment by GNE-272 in BxPC-3 and PANC1. **C** The relative mRNA expression of SNAI2 was upregulated after EGR1 overexpression and reduced to the baseline after further treatment with GNE-272. **D** The expression of SNAI2 protein were upregulated after EGR1 overexpression and significantly reduced after further treatment with GNE-272. **E** The blot image of Co-IP assay showed that EGR1 directly interacted with p300 and CBP. **F** The blot image of Co-IP assay showed that p300 directly interacted with EGR1. **G** The results of ChIP-qPCR showed that the combination of EGR1 and SNAI2 promoter regions was downregulated after treatment with GNE-272. *: p < 0.05; ***: p < 0.001

P300/CBP is considered a transcriptional coactivator that function in transcriptional initiation [17]. Previous studies have demonstrated that EGR1 interacted with the coactivator proteins p300/CBP to stimulate gene transcription in specific cell lines [18–20]. Thus, we wondered whether p300/CBP was involve in the transcription regulation of EGR1 on the SNAI2 promoter in pancreatic cancer. First, we utilized GNE-272, a potent and selective p300/CBP inhibitor [21], to examine the effect of p300/CBP on SNAI2 expression. The results showed that, after treatment with GNE-272, the cell migration and invasion ability were apparently impaired (Additional file 4: Fig. S3A) and the expression of SNAI2 was downregulated, both at mRNA level and protein level (Fig. 6A, B). Besides, after treatment with GNE-272, the EMT-related proteins were also altered (Additional file 4: Fig. S3B). EP300 siRNA was also employed to block the mRNA expression of p300 and after knockdown of p300, the mRNA and protein expression of SNAI2 was downregulated (Additional file 4: Figure S3C–E). The GNE-272 or EP300 siRNA was added after overexpression of EGR1, it can be seen that the p300 expression was downregulated and the expression of SNAI2 was correspondingly downregulated at both mRNA level and protein level (Fig. 6C, D) (Additional file 4: Fig. S3F). These results indicated that reduction of p300/CBP significantly affected the expression of SNAI2, both at mRNA level and protein level. Further, the results of Co-IP assay verified the direct protein interaction of EGR1 and p300/CBP, using EGR1 antibody and p300 antibody, respectively (Fig. 6E, F). Finally, ChIP-qPCR assay was performed. After the addition of GNE-272 or knockdown of EP300, the promoter activity of SNAI2 was downregulated (Fig. 6G and Additional file 4: Fig. S3G). These results demonstrated that EGR1 formed a complex with p300/CBP, thus initiated the transcription of SNAI2 gene.

### EGR1 promoted liver metastasis of pancreatic cancer in vivo

**Fig. 7:**
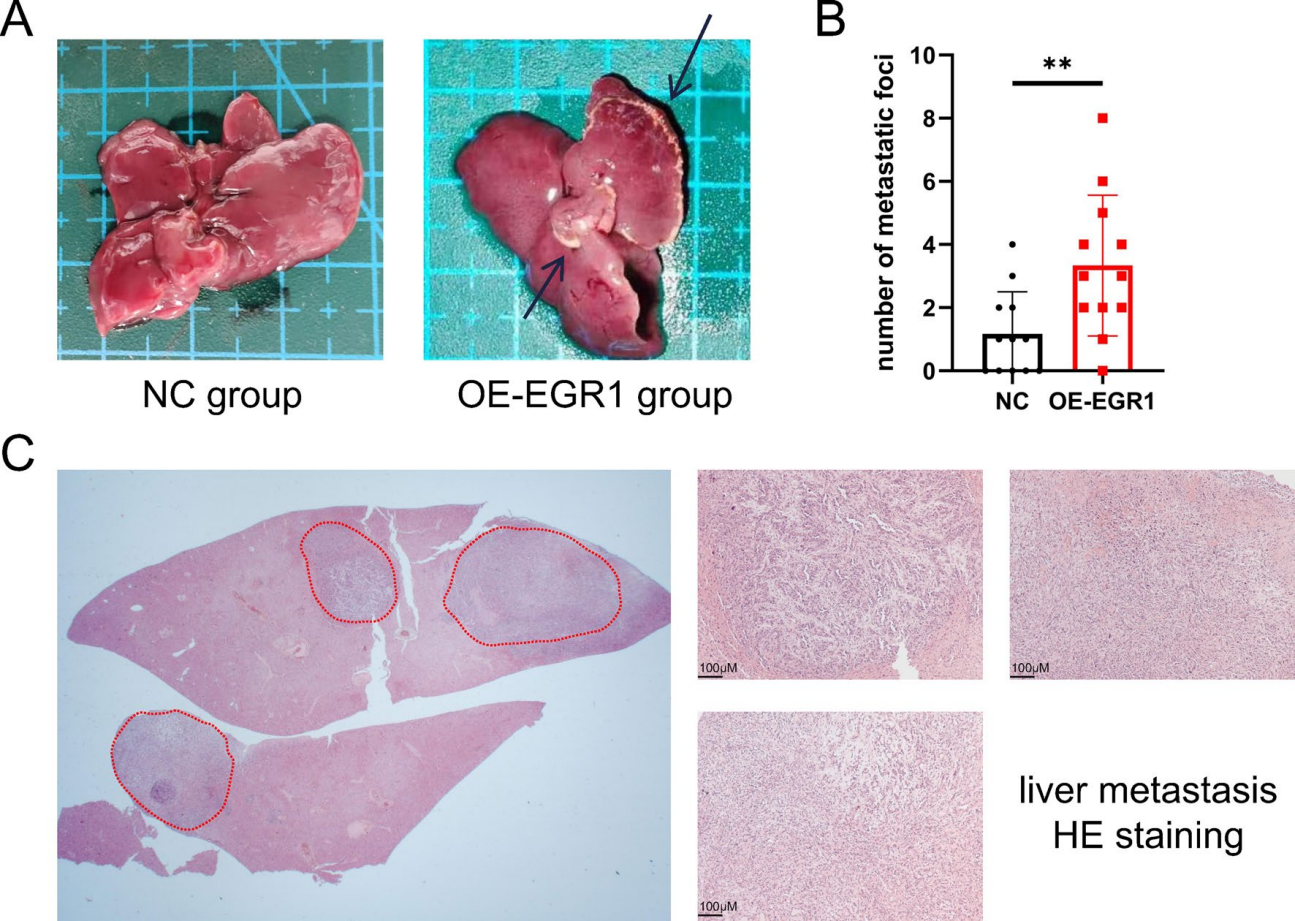
EGR1 promoted liver metastasis of pancreatic cancer in vivo. **A** The representative pictures of liver in normal control group and EGR1-overexpression group. The arrows showed probable liver metastatic foci of pancreatic cancer. **B** The statistical chart of the number of liver metastatic foci in each group. **C** The representative pictures of liver metastatic foci in HE staining

In order to further verified the role of EGR1 in pancreatic cancer in vivo, normal control PANC1 cells and stable EGR1-overexpressed PANC1 cells were injected into the spleen of nude mice. After four weeks of feeding, the mice were sacrificed and dissected. 12 mice for each group were involved in statistical analysis. Compared with the normal control group, the EGR1-overexpression group has more liver metastatic colonies (Fig. 7A, B). The typical HE staining of metastatic foci was shown in Fig. 7C. These results demonstrated that EGR1 promoted liver metastasis of pancreatic cancer in vivo.

## Discussion

EGR1 is generally expressed in many types of tissues and involved in vital physiological processes, such as cell differentiation, migration, growth and apoptosis, etc. [22–24]. Cellular EGR1 expression could be stimulated by growth factors, reactive oxygen species (ROS), hypoxia or other stimuli [25–27] and activated EGR1 could either initiate or inhibit the transcription of its target genes via its Cys2-His2 zinc finger composition. In the field of cancer research, EGR1 functioned as both a tumor promoter and a tumor suppressor. In prostate cancer, EGR1 could upregulate angiogenic and osteoclastogenic factors, including PDGFA, IL6, IL8, etc. and thus promote cancer metastasis [28]. In gastric cancer, EGR1 promoted the transcription of linc01503 and further affected the cell cycle [29]. EGR1 also suppressed colon cancer progression by a PTEN-EMT pathway [30], which seems to contradict our study. In pancreatic cancer, EGR1 contributed to pancreatic tumorigenesis and inflammation-induced epithelial cell reprogramming as previously described [31].

In the present study, we identified EGR1 as a pro-metastasis factor in pancreatic cancer cells, which promoted cell migration and invasion via the SNAI2-EMT pathway. SNAI2 or Slug/Snail2 serves as one of the core EMT-TFs including ZEB1, ZEB2, SNAI1, SNAI2 and TWIST, which could particularly repress epithelial markers such as E-cadherin and activate mesenchymal markers such as Vimentin and N-cadherin, etc. [32]. Therefore, when we found the relationship between EGR1 and EMT, we immediately thought of the possibility of EGR1 regulating these TFs. In addition to the regulation of EMT, SNAI2 also conferred resistance to MEK1/2 inhibitors and gemcitabine in pancreatic cancer, as previously reported [33, 34]. While, our research did not focus on the relationship between EGR1 and drug sensitivity which could be investigated later, we studied the role of EGR1 in EMT pathway. Transcriptional regulation involves not only the direct binding of TF to regulatory elements of target genes but also the complex interactions between TF and TF-binding proteins including cofactors, mediators, TF activity modulating enzymes, etc. [35, 36]. The formation of transcription complexes is necessary for the regulation of the DNA accessibility to allow chromatin to open, so as to achieve gene transcription [37]. We thus wonder whether EGR1 activated the transcription of SNAI2 through forming complexes with cofactors. Coincidentally, the string database (https://string-db.org) has shown the potential combination of EGR1 and p300/CBP, which has been further verified in our research.

P300/CBP has multiple regulatory mechanisms, among which transcription coactivation is the basal machinery, that p300/CBP could form a complex with transcription factor and improve the synergy of transcription [38]. Previously, Wu et al. found that under ethanol exposure, EGR1 could combine with p300/CBP and activate the transcription of angiotensin-converting enzyme (ACE) [18], which supported our conclusion. Besides, p300/CBP serves as histone acetyltransferases (HATs) that can acetylate histones and remodel chromatin to activate gene transcription [38]. Inhibition of p300/CBP changed the acetylation of H3K18 and H3K27, thus attenuating the transcription of glycolysis-related metabolic enzymes, and retarded the hepatocellular carcinoma progression [39]. While p300/CBP could form dynamic aggregates to coordinate the above two functions, and TF/p300 co-condensation contributes to transcriptional burst regulation and synergistic gene control [40].

There are still limitations in this study. First, within the scope of scientific research, the role of EGR1 was not uniform across all types of cancer. As described above, EGR1 showed anti-tumor activity in colon cancer, hepatocarcinoma, leukemia, human fibrosarcoma, etc. [13–15, 30], which was not consistent with our study. We believe this may be due to the tissue and cell specificity. Second, within the EMT pathway, we thought the frequent indicators we detected in this study were sufficient but imperfect. There are more of those, such as P-cadherin, also named CDH3, which was also a classical cell adhesion molecule. However, unlike traditional cadherins (E-cadherin and N-cadherin), P-cadherin is a double-edged sword in cancer pathology, and its behavior in malignant environments is clearly dependent on the cellular context [41]. Thus, it was not included in our research. Besides, the EMT was considered a continuous, dynamic and reversible process, resulting in cancer cells showing a variety of intermediate or partial states, called epithelial-mesenchymal plasticity (EMP) [42]. With the development of novel sequencing technology, the study of EGR1 would be much more precise.

## Conclusion

In this research, we revealed that EGR1 promoted pancreatic cancer metastasis both in vitro and in vivo. Further, EGR1 promoted EMT process via transcription of SNAI2. P300/CBP that interacts with EGR1 stabilized the transcription complex. Blocking EGR1-SNAI2 pathway might be a novel anticancer strategy in pancreatic cancer.

## Supplementary Information

**Additional file 1: Table S1.** Primer sequences in this research were listed below.

**Additional file 2: Figure S1.** (A) The results of wound healing assay showed impaired cell motility after knockdown of EGR1. (B) The results of wound healing assay showed enhanced cell motility after overexpression of EGR1. (C) The predicted RNA correlation of EGR1 and TWIST1 in pancreatic cancer. (D) The ChIP-qPCR results of EGR1 and SNAI1 promoter. (E) The effect of SNAI2 overexpression and kncokdown were verified by qRT-PCR and western blot assays. *: p < 0.05; ***: p < 0.001.

**Additional file 3: Figure S2.** (A) The cell migration and invasion ability were downregulated after knockdown of SNAI2 in PANC1. (B) The cell migration and invasion ability were upregulated after overexpression of SNAI2 in PANC1. (C) The fluorescence intensity of E-cadherin was upregulated after knockdown of SNAI2 in BxPC-3 and downregulated after overexpression of SNAI2 in PANC1. ***: p < 0.001.

**Additional file 4: Figure S3.** (A) The cell migration and invasion ability were diminished after treatment with GNE-272 in PANC1. (B) The expression of EMT-related proteins was downregulated after treatment with GNE-272 in PANC1. (C) and (D) The mRNA expression of SNAI2 was downregulated after knockdown of EP300 by qPCR analysis in BxPC-3 and PANC1. (E) The expression of SNAI2 was downregulated after knockdown of EP300 by western blot analysis in BxPC-3 and PANC1. (F) The expression of SNAI2 was upregulated after EGR1 overexpression and further downregulated after EP300 knockdown by western blot analysis in PANC1. (G) The combination of EGR1 and SNAI2 promoter was impaired after knockdown of EP300 in BxPC-3. *: p < 0.05; **: p < 0.005; ***: p < 0.001.

## Data Availability

Any individual or group may contact the corresponding author to obtain relevant research data.

## Abbreviations

EMT: Epithelial-mesenchymal transition
EGR1: Early growth response 1
SRB: Sulforhodamine B
IHC: Immunohistochemistry
IF: Immunofluorescence
OS: Overall survival
GSEA: Gene set enrichment analysis
