# Supplementary material for: EGR1 induces EMT in pancreatic cancer via a P300/SNAI2 pathway
Source: J Transl Med. 2023 Mar 17;21:201. doi: 10.1186/s12967-023-04043-4 (PMC10021983; doi:10.1186/s12967-023-04043-4)
Supplement: Supplementary file 1 — Additional file 1: Table S1. Primer sequences in this research were listed below. [file 12967_2023_4043_MOESM1_ESM.docx]

Additional Table S1. Primer sequences in this research were listed below.

| **Primer Name** | **Sequence** |
| --- | --- |
| β-actin forward | TGGCACCCAGCACAATGAA |
| β-actin reverse | CTAAGTCATAGTCCGCCTAGAAGCA |
| EGR1 forward | ACGAGAAGGTGCTGGTGGAGAC |
| EGR1 reverse | GAGATGGTGCTGAGGACGAGGAG |
| Vimentin forward | CCTTCGTGAATACCAAGACCTGCTC |
| Vimentin reverse | AATCCTGCTCTCCTCGCCTTCC |
| Snail1 forward | TCAGATGAGGACAGTGGGAAAGGC |
| Snail1 reverse | ACTGAAGTAGAGGAGAAGGACGAAGG |
| SNAI2 forward | CTGTGACAAGGAATATGTGAGC |
| SNAI2 reverse | CTAATGTGTCCTTGAAGCAACC |
| ZEB1 forward | AGTGTTACCAGGGAGGAGCAGTG |
| ZEB1 reverse | TTTCTTGCCCTTCCTTTCCTGTGTC |
| ZEB2 forward | GAAGACAGAGAGTGGCATGTAT |
| ZEB2 reverse | GTGTGTTCGTATTTATGTCGCA |
| TWIST1 forward | GACTTCCTCTACCAGGTCCTCCAG |
| TWIST2 reverse | TCCAGACCGAGAAGGCGTAGC |
| SNAI2-promoter -96~-83 forward | TCCAATCACAGCTGAGAGGTTC |
| SNAI2-promoter -96~-83 reverse | ACAGCCCATTTTGAACCAGA |
| SNAI2-promoter -499~-486 forward | GCATCTGGAGAGGTTTGCCTT |
| SNAI2-promoter -499~-486 reverse | GAGTCCCAGGAGAGCGTCC |
| SNAI2-promoter -625~-612 forward | CTACTCAGGGCTTCCGCGA |
| SNAI2-promoter -625~-612 reverse | TCCGATCAGCCTGCCTTTAG |
| SNAI1-promoter forward | GCTTCCTCCCCAGTGATGTG |
| SNAI1-promoter reverse | GGACACCTGACCTTCCGACG |
