# Supplementary figures and images for: EGR1 induces EMT in pancreatic cancer via a P300/SNAI2 pathway
Source: J Transl Med. 2023 Mar 17;21:201. doi: 10.1186/s12967-023-04043-4 (PMC10021983; doi:10.1186/s12967-023-04043-4)

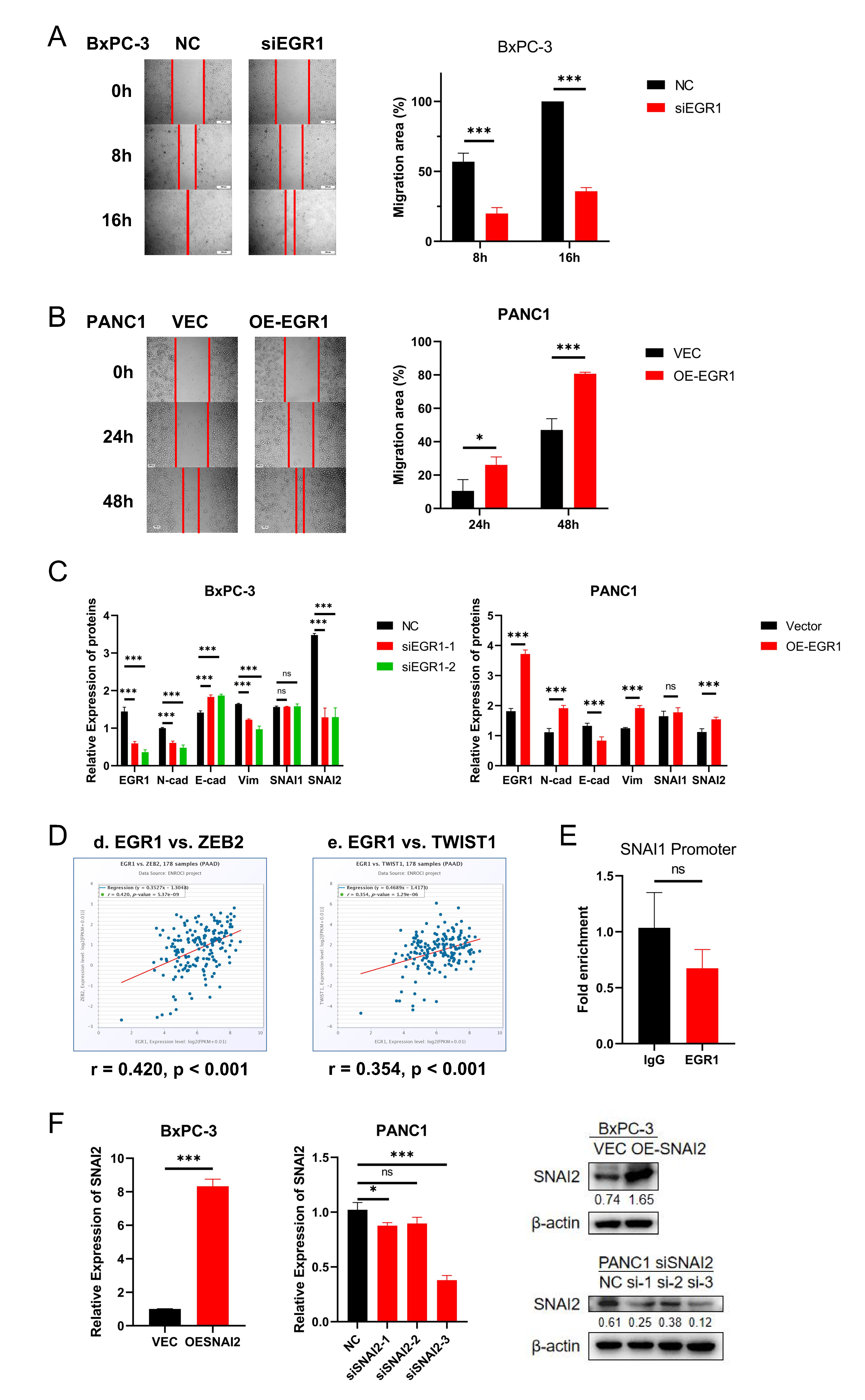

Supplement: Supplementary file 2 — Additional file 2: Figure S1. (A) The results of wound healing assay showed impaired cell motility after knockdown of EGR1. (B) The results of wound healing assay showed enhanced cell motility after overexpression of EGR1. (C) The predicted RNA correlation of EGR1 and TWIST1 in pancreatic cancer. (D) The ChIP-qPCR results of EGR1 and SNAI1 promoter. (E) The effect of SNAI2 overexpression and kncokdown were verified by qRT-PCR and western blot assays. *: p < 0.05; ***: p < 0.001. [file 12967_2023_4043_MOESM2_ESM.tif]

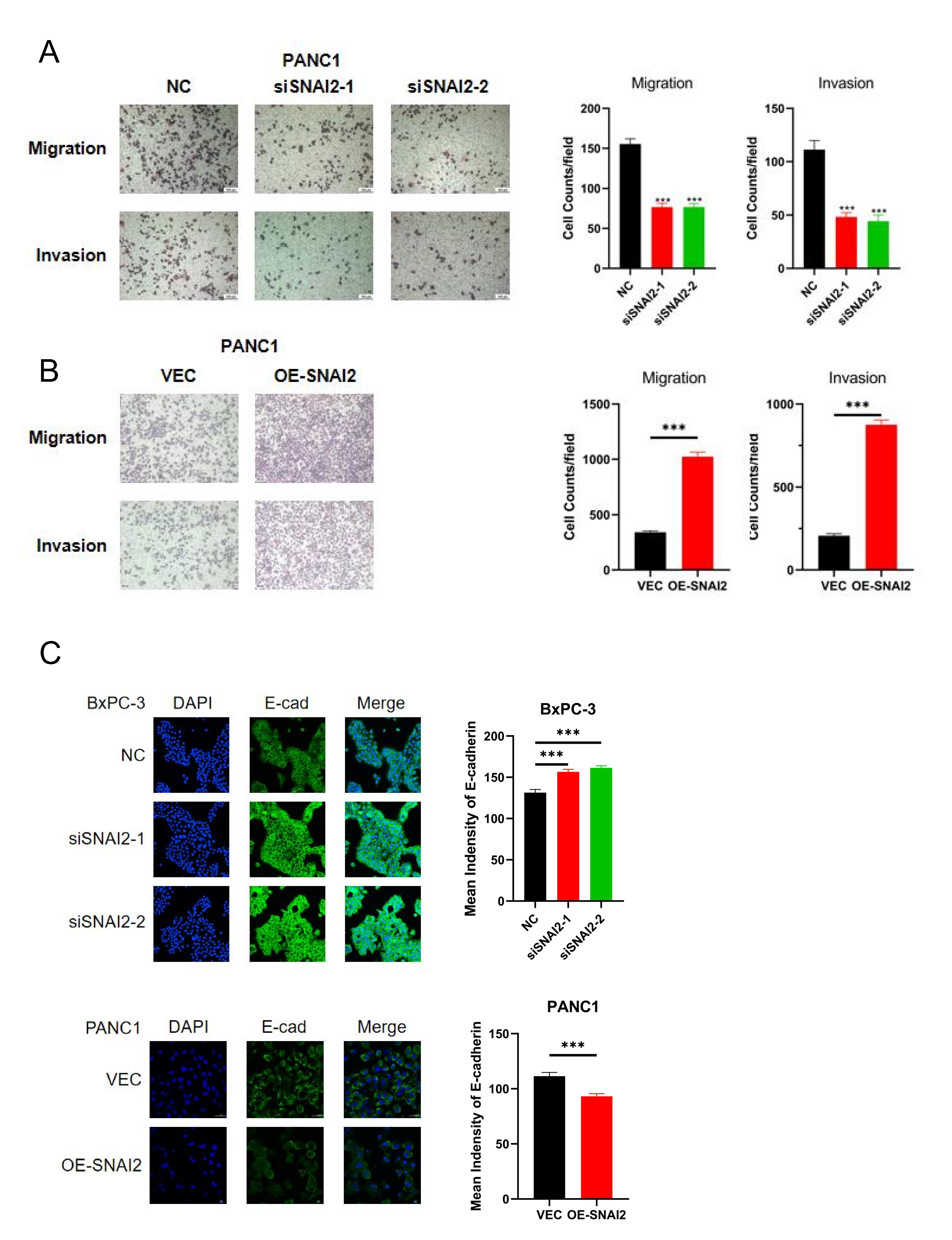

Supplement: Supplementary file 3 — Additional file 3: Figure S2. (A) The cell migration and invasion ability were downregulated after knockdown of SNAI2 in PANC1. (B) The cell migration and invasion ability were upregulated after overexpression of SNAI2 in PANC1. (C) The fluorescence intensity of E-cadherin was upregulated after knockdown of SNAI2 in BxPC-3 and downregulated after overexpression of SNAI2 in PANC1. ***: p < 0.001. [file 12967_2023_4043_MOESM3_ESM.tif]

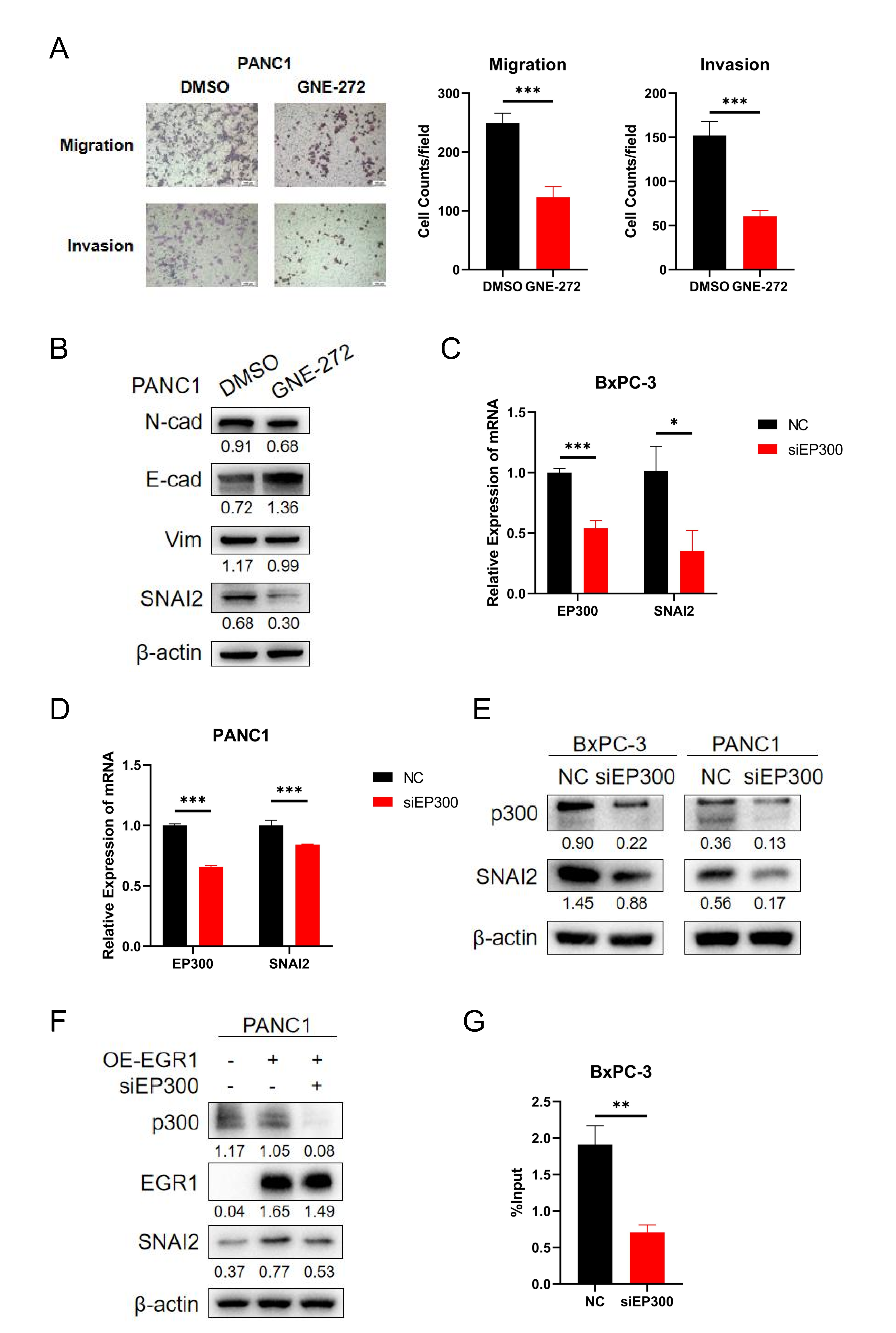

Supplement: Supplementary file 4 — Additional file 4: Figure S3. (A) The cell migration and invasion ability were diminished after treatment with GNE-272 in PANC1. (B) The expression of EMT-related proteins was downregulated after treatment with GNE-272 in PANC1. (C) and (D) The mRNA expression of SNAI2 was downregulated after knockdown of EP300 by qPCR analysis in BxPC-3 and PANC1. (E) The expression of SNAI2 was downregulated after knockdown of EP300 by western blot analysis in BxPC-3 and PANC1. (F) The expression of SNAI2 was upregulated after EGR1 overexpression and further downregulated after EP300 knockdown by western blot analysis in PANC1. (G) The combination of EGR1 and SNAI2 promoter was impaired after knockdown of EP300 in BxPC-3. *: p < 0.05; **: p < 0.005; ***: p < 0.001. [file 12967_2023_4043_MOESM4_ESM.tif]
